# List of Accidents, Admitted into the Pennsylvania Hospital, from Sept. 5th, to Sept. 19th, 1838

**Published:** 1838-09-26

**Authors:** J. Forsyth Meigs

**Affiliations:** Resident Surgeon


### PENNSYLVANIA HOSPITAL.

*List of Accidents, admitted into the Pennsylvania Hospital, from Sept. 5th, to Sept. 19th, 1838.*

[Reported by J. Forsyth Meigs, M. D., Resident Surgeon.]

A case of severe contusion of the ankle, with a lacerated wound on the inside of the foot and upper part of the leg, involving merely the integuments, caused by the leg's being jammed in between two rail-road cars. When the man was brought in, there was great swelling about the foot, with severe pain and inflammation. The leg was placed at rest, and lead water applied; the man has since been attacked with symptoms of mania-a-potu, which were relieved by assafœtida and opium; at present, the wound is suppurating and doing well; dressed with poultice and the fracture-box.—A case of slight concussion of the brain, with fracture of the left clavicle, from a fall down a hatchway.

Some fever arose, for which the patient was bled and purged; the fracture is treated with the clavicle apparatus, and the patient is now doing well.—A case of fracture of the humerus, within the anatomical neck, from a fall: the patient was admitted several days after the accident, when he complained of severe pain about the shoulder; a crepitus was detected upon placing the hand in the axilla, and rotating the arm—also, by grasping the head of the bone. Dressed with an angular splint on the inside of the arm, extending from the axilla to the fingers, and two short splints, behind and before, with the arm brought close to the side, by which means the fracture is retained perfectly in place; doing well.—A case of fracture of the fibula of the right leg, several inches above the ankle; treated in the usual way; doing well.—A case of gunshot wound of the hand, a load of shot having passed through the palm, separating the middle metacarpal bones, and dividing the superficial palmar arch: hæmorrhage not great, owing to the cauterization by the powder; one vessel secured; strips of adhesive plaster and lint applied; arm placed on splint, elevated, and lead water kept to it by a syphon; doing well.—A case of fracture of the olecranon process of the ulna, from a fall from the second story of a house; when admitted, two days after the accident, the whole arm was very much swollen and inflamed, with great tenderness; the arm being flexed was laid on a splint, leeched, elevated, and cold applied, which reduced the swelling in a few days; at present, the arm is extended; a figure of eight bandage applied round the elbow, with a straight splint on the front of the arm; doing well. —A case of lacerated wound of the scalp, over the left parietal bone, from a fall; one of the temporal arteries was wounded; hæmorrhage at first arrested by pressure, but returning, the artery was secured by a ligature; patient attacked with mania-a-potu, for which he took opiates and stimulants, and had a blister on the back of the neck; since dead.—A case of fracture of both bones of the right leg, about two inches above the ankle, with three small lacerated wounds, from the bite of a dog: leg placed in fracture-box, and, as considerable oozing of blood took place, bran was placed about it, which arrested the hæmorrhage; patient since attacked with mania-a-potu, for which he is now under treatment.—A case of lacerated wound of the scalp, over the occiput, from a fall from a height. When admitted, there was profuse hæmorrhage from the left occipital artery, which, from its great retraction, and the density of the aponeurosis, it was found impossible to secure by the tenaculum; pressure was resorted to, but without success; the hæmorrhage was effectually arrested by the application of the hare-lip suture; since attacked with mania-a-potu, which has been cured by the use of opiates, and counter-irritation to the back of the neck; the wound has united by the first intention.—A case of severe sprain of the left ankle, from a fall: when admitted, there was much inflammation and swelling, which has been removed by rest and cold applications.

The case of contusion of the ankles mentioned in last report, has been discharged cured.—The case of contusion of the scalp has been discharged cured, having had no bad symptoms since the last report.—The case of partial fracture of the leg, has since then been treated by a straight splint on the inside of the leg, by which means the deformity is almost entirely removed.

